# Supplementary figures and images for: Vitamin B12 is neuroprotective in experimental pneumococcal meningitis through modulation of hippocampal DNA methylation
Source: J Neuroinflammation. 2020 Apr 1;17:96. doi: 10.1186/s12974-020-01763-y (PMC7115084; doi:10.1186/s12974-020-01763-y)

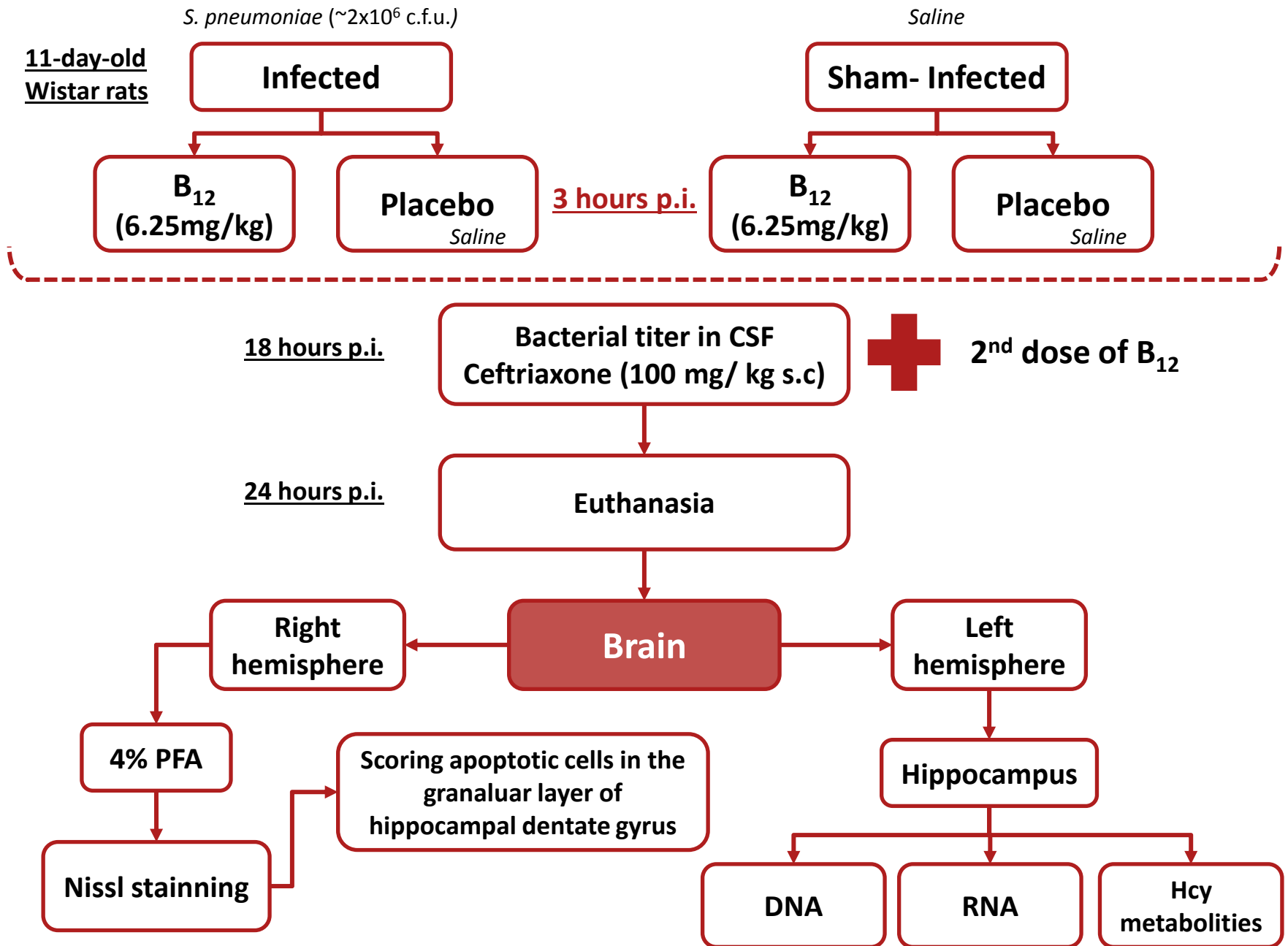

Supplement: Supplementary file 1 — Additional file 1. Experimental design. Diagrammatic representation of the experimental design of this study. [file 12974_2020_1763_MOESM1_ESM.pdf]
