# Supplementary material for: Vitamin B12 is neuroprotective in experimental pneumococcal meningitis through modulation of hippocampal DNA methylation
Source: J Neuroinflammation. 2020 Apr 1;17:96. doi: 10.1186/s12974-020-01763-y (PMC7115084; doi:10.1186/s12974-020-01763-y)
Supplement: Supplementary file 2 — Additional file 2. Effects of BM and adjuvant therapy with vitamin B12 on gene expression in the hippocampus. Table showing the expression patterns of 11 genes previously implicated in the pathophysiology of pneumococcal meningitis that were not regulated by adjuvant therapy with vitamin B12. [file 12974_2020_1763_MOESM2_ESM.docx]

**Additional file 2**: Effects of BM and adjuvant therapy with vitamin B_12_ on gene expression in the hippocampus.

|  | **Relative expression in groups** | | | |  | ***P*-values (2-way ANOVA)** | | |
| --- | --- | --- | --- | --- | --- | --- | --- | --- |
| **Genes** | **Sh-Sal** | **Sh-B_12_** | **Infect-Sal** | **Infect-B_12_** |  | **Effect of meningitis** | **Effect of vitamin B_12_** | **Interaction** |
| ***Mmp9*** | 0.0039±0.002 | 0.0061±0.003 | 0.0424±0.014 | 0.0409±0.0409 |  | < 0.001 | 0.9684 | 0.8385 |
| ***Tjp2*** | 0.0368±0.005 | 0.0737±0.033 | 0.1025±0.028* | 0.0926±0.012 |  | 0.0017 | 0.2419 | 0.0517 |
| ***Ocln*** | 0.0250±0.008 | 0.0271±0.008 | 0.0166±0.006 | 0.0120±0.005* |  | < 0.001 | 0.6498 | 0.2491 |
| ***Timp1*** | 0.0272±0.009 | 0.0139±0.004 | 1.251±0.791* | 0.9384±0.441* |  | < 0.001 | 0.4247 | 0.4628 |
| ***Ccl2*** | 0.0019±0.09 | 0.0027±0.002 | 2.165±0.344* | 2.163±0.439* |  | < 0.001 | 0.7075 | 0.7075 |
| ***Il-6*** | 0.0002±0.0001 | 0.0002±0.0001 | 0.0513±0.012* | 0.0337±0.003 |  | < 0.001 | 0.4040 | 0.4039 |
| ***Casp3*** | 0.1472±0.070 | 0.1619±0.063 | 0.0828±0.034 | 0.0991±0.042 |  | 0.0068 | 0.4709 | 0.9691 |
| ***Nfκβ*** | 0.0711±0.035 | 0.0436±0.013 | 0.1122±0.059 | 0.1712±0.104* |  | 0.0045 | 0.5621 | 0.1200 |
| ***Tnfa*** | 0.0019±0.001 | 0.0014±0.001 | 0.0355±0.017* | 0.0345±0.009* |  | < 0.001 | 0.8554 | 0.9583 |
| ***Il-10*** | 0.0001±0.001 | 0.00004±0.00004 | 0.0084±0.010 | 0.0029±0.003 |  | 0.0125 | 0.1941 | 0.1989 |
| ***Cxcl1*** | 0.0003±0.0001 | 0.0003±0.0001 | 0.4954±0.148* | 0.2427±0.051 |  | 0.0017 | 0.2373 | 0.2371 |

Data are expressed as means ± S.D. Statistical differences were assessed using a 2-way ANOVA (Bonferroni post-hoc test) to examine the effects of meningitis and adjuvant therapy with vitamin B_12_.* denotes statistically significant differences (*P* < 0.05) at Bonferroni post-hoc test when compared with the respective sham-infected control (Sh-Sal or Sh-B_12_). Sh-Sal, sham-infected + saline; Sh-B_12_, sham-infected + vitamin B_12_; Infect-Sal, infected + saline; Infect-B_12_, infected + vitamin B_12_.
